# Appointments and Their Confirmation

**Published:** 1913-07-12

**Authors:** 


### APPOINTMENTS AND THEIR CONFIRMATION.

By "HUMERAL CONDYLE."

The administrative acts of the Local Government Boards are at times astonishing. The powers of the Board are allowed to be dormant till their existence is forgotten, when suddenly and without warning the Board is galvanised into action, and, sad to relate, too often with ludicrous results. The Peamount Sanatorium incident is an excellent example of this vacillating type of administration. The Sanatorium was recognised for Insurance benefit; the post of medical superintendent was duly advertised, not only in local papers, but in all the medical journals. The committee appointed a candidate whose qualifications fulfil all the requirements for tuberculosis officers as prescribed by the Local Government Board's regulations. Next morning a communication was received from the Irish Local Government Board expressing surprise, and threatening to cancel the recognition of this Dublin Sanatorium if the appointment was persisted in. The British Medical Association in Dublin supports the committee and have protested against this futile objection to a well-qualified candidate.

What is the attitude of the English Board in similar circumstances? Fortunately, a striking case is within our knowledge. The Metropolitan Asylums Board were permitted to utilise the Downs School, now known as the Downs Sanatorium, as a sanatorium for insured consumptives. The post of medical superintendent was not even advertised. The position was given to an officer of the fever service of the Metropolitan Asylums Board. Perhaps the English Board will tell the Irish Board how much experience in tuberculosis this officer possessed? Patronage critics were fobbed off temporarily by the pretence that the appointment was probationary for one year; the inner circle knew that it would be permanent after this brief interval. The English Local Government Board sanctioned such an appointment without objection or comment. Perhaps the committee of the Dublin Peamount Sanatorium should follow this dodge of circumventing the official objection by making the appointment only for a year.

A consideration of this incident leads to a brief review of all appointments without advertisements. In the case of the Downs Sanatorium the junior officers were also recruited from the fever service. These, however, had a limited experience in tuberculosis. There were no advertisements, not even a circular to the other services of the Metropolitan Asylums Board with tuberculosis experience.

The Local Government Board is supposed to cast a watchful eye on appointments without advertisements. A certain degree of patronage will always exist in every appointment, except those by competitive examination, but publicity is a great deterrent to patronage and its abuse. Some guarantee as to the "purity" of appointments is offered by the wide advertising of the vacancy with a strict observance of the disqualification penalty for canvassing. Great laxity is, however, allowed to public bodies in the election of officers.

The regulations of the Local Government Board that appointments to non-advertised vacancies must be exceptional, and only with their previous consent, are forgotten when such a policy is convenient. This knowledge emboldens even elected bodies to shirk the advertising of vacancies. It is done on the ground of economy. Instances are becoming more and more frequent.

The Sheffield Council appoint an assistant medical officer of health without advertisement. The Wandsworth Guardians appoint a deputy medical superintendent at £350 to £400 without advertising. The question naturally arises: are such appointments fair to the men who have no friends in the inner circle of the appointing bodies?

The Metropolitan Asylums Board need a research pathologist. Instead of a public advertisement, the Managers issue a semi-private account of the terms to the pathological departments of the British Universities and medical schools and some foreign ones.

In the case of an institution the promotion of one of the juniors or many of the juniors "one up" on the resignation of the senior is quite legitimate, because in these cases the choice is the result of the medical superintendent's advice. But the highest posts should always be filled in the glare of a public advertisement.

Some democrats are loud in condemnation of patronage appointments in the Civil Service. It is an open accusation that a gross abuse of patronage is a common feature of Civil Service appointments and promotions, and that such a system could only obtain in Government departments untouched by ordinary public criticism. But if the head of a Government department cannot judge the wisdom of futility of certain possible promotions, it is obvious that lay public bodies can have no defence for certain "selected" appointments without advertisement.

